# Adaptive Fuzzy Control for Uncertain Fractional-Order Financial Chaotic Systems Subjected to Input Saturation

**DOI:** 10.1371/journal.pone.0164791

**Published:** 2016-10-26

**Authors:** Chenhui Wang

**Affiliations:** College of Applied Mathematics, Xiamen University of Technology, Xiamen 361024, China; Lanzhou University of Technology, CHINA

## Abstract

In this paper, control of uncertain fractional-order financial chaotic system with input saturation and external disturbance is investigated. The unknown part of the input saturation as well as the system’s unknown nonlinear function is approximated by a fuzzy logic system. To handle the fuzzy approximation error and the estimation error of the unknown upper bound of the external disturbance, fractional-order adaptation laws are constructed. Based on fractional Lyapunov stability theorem, an adaptive fuzzy controller is designed, and the asymptotical stability can be guaranteed. Finally, simulation studies are given to indicate the effectiveness of the proposed method.

## 1 Introduction

Thirty years ago, Stutzer, an economist, first obtained the chaotic behavior in financial system [1]. Up to now, it has been shown that financial systems can exhibit complicated behavior, for example, chaos [2–5]. Besides, financial crisis can be seen as a kind of chaotic phenomenon [2, 6]. Meanwhile, researchers have found that system uncertainties in the economic development, for example, the abrupt change variety of economy in frequency and the influence of non-financial elements, are increasing [7–10]. Accordingly, taking the chaotic behavior as well as system uncertainties into consideration, to solve financial crisis and some relevant problems, it is advisable to study the chaos control methods for financial systems.

Fractional calculus is an old topic which has a history more than 300 years. Now, it can be seen in many domains, ranging from life sciences and materials engineering to secret communication and control theory [11–21]. One of a major merits of fractional-order systems, compared with the integer ones, is that the fractional-order ones have memory, and they have been proven to be a powerful technique to describe the hereditary and memory properties of a lot of materials and processes. It is known that financial variables possess memories, so fractional-order models can be well used to describe dynamical behaviors in economic systems. Up to now, some work has been done to control or discuss the dynamical behavior of fractional-order financial systems [4, 9, 10, 22–28]. Generally speaking, the control and synchronization for fractional-order financial chaotic systems is becoming a hot research area.

On the other hand, most of actual physical systems may be inflicted by input saturation because there exist a limited size of actuators, sensors, and interfacing system devices [29–33]. These input constraints usually damage the performance of the system or cause system’s ultimate instability if it is not well handled. With respect to integer-order systems with input saturation, many control methods have been given (for example, see, [29–36]). The usually used idea to handle input saturation is that the sector bounded conditions are associated with input nonlinearities, thus the stability of the system can be discussed based on Lyapunov stability criterion. However, as a generalization of integer-order system, fractional-order systems have many very different properties. Thus, these methods which are effective for integer-order systems can not be used to control fractional-order system directly. Up to now, there are only very few literatures which consider the controller design for fractional-order nonlinear systems with input saturation [37, 38]. In above prior work, the input saturation is handled by some linear inequality, which contains a restricted condition. As far as financial systems are considered, the input saturation phenomenon may occur naturally. So, how to design effective controllers for fractional-order financial chaotic systems is a challenging and meaningful work.

Motivated by above discussions, in this paper, we will study the control for uncertain fractional-order financial chaotic system subjected to input saturation with system uncertainty and external disturbance. Our contributions consist in: (1) control of fractional-order financial chaotic system subjected to input saturation is considered in this paper, and the input saturation is handled by a kind of transformation; (2) Fuzzy logic systems are used to approximate nonlinear functions which contain both system uncertainties and input saturation, and an adaptive fuzzy controller is proposed by using the fractional Lyapunov method; (3) to update the fuzzy parameters, fractional-order adaptation laws, which have one more free degree compared with the conventional integer-order ones, are designed.

The remainder of this paper is organized as follows: Section 2 lists some preliminaries. In Section 3, adaptive fuzzy control algorithm is given and stability of the closed-loop system is analyzed. Simulation studies are presented in Section 4. Finally, Section 5 gives the conclusions of this work.

## 2 Preliminaries

### 2.1 Preliminaries

The fractional-order integrodifferential operator can be seen as a extended concept of the integer-order one. The mostly commonly utilized definitions in literature are Grünwald-Letnikov, Riemann-Liouville, and Caputo definitions. The main reason why Caputo’s derivative is introduced for engineering applications consists in that, just like in integer-order systems, its Laplace transform requires integer-order derivatives for the initial conditions. On the contrary, the Laplace transform of the Riemann-Liouville definition contains fractional-order derivatives that are difficult to be physically interpreted. The Caputo’s derivative will be used in this paper. The *q*-th fractional integral can be given as

$$\begin{array}{c} I^{-q}f\left(t\right)=\frac{1}{\Gamma (q)}\int_{0}^{t}\frac{f(\tau )}{{\left(t-\tau \right)}^{1-q}}d\tau , \end{array}$$ (1)

where Γ(⋅) stands for the Gamma function.

The *q*-th fractional-order derivative is given as

$$\begin{array}{c} D^{q}f\left(t\right)=\frac{1}{\Gamma (n-q)}\int_{0}^{t}\frac{f^{\left(n\right)}\left(\tau \right)}{{\left(t-\tau \right)}^{q+1-n}}d\tau , \end{array}$$ (2)

where *n* − 1 ≤ *q* < *n* (n∈N). In this paper, only the case 0 < *q* ≤ 1 is included.

To facilitate the controller design, let us give the following results first.

**Definition 1** [11]. *The Mittag-Leffler function can be given as*

$$\begin{array}{c} E_{\alpha_{1},\alpha_{2}}\left(\zeta \right)=\sum_{k=0}^{\infty }\frac{\zeta^{k}}{\Gamma (\alpha_{1}k+\alpha_{2})}, \end{array}$$ (3)

*where* *α*_1_ *and* *α*_2_ *are positive constants, and* ζ∈C.

The Laplace transform of Eq (3) is [11]

$$\begin{array}{c} L\left\{t^{\beta -1}E_{\alpha_{1},\alpha_{2}}\left(-at^{\alpha_{1}}\right)\right\}=\frac{s^{\alpha_{1}-\alpha_{2}}}{s^{\alpha_{1}}+a}. \end{array}$$ (4)

**Lemma 1** [11]. *Let* $\alpha_{2}\in C$. *If* 0 < *α*_1_ < 2 *and* $\frac{\pi \alpha_{1}}{2}<\iota <\min\left\{\pi ,\pi \alpha_{1}\right\}$, *then, when* |*ζ*|→∞ *and* *ι* ≤ |arg(*ζ*) ≤ *π*, *we have*:

$$\begin{array}{c} E_{\alpha_{1},\alpha_{2}}\left(\zeta \right)=-\sum_{j=1}^{n}\frac{1}{\Gamma \left(\alpha_{2}-\alpha_{1}j\right)\zeta^{j}}+o(\frac{1}{{\left|\zeta \right|}^{n+1}}). \end{array}$$ (5)

**Lemma 2** [11]. *Let* 0 < *α*_1_ < 2 *and* $\alpha_{2}\in R$. *If* $\frac{\pi \alpha_{1}}{2}<\iota \leq \min\left\{\pi ,\pi \alpha_{1}\right\}$, *then we can obtain*

$$\begin{array}{c} |E_{\alpha_{1},\alpha_{2}}\left(\zeta \right)|\leq \frac{C}{1+|\zeta |} \end{array}$$ (6)

*where* *C* > 0, *ι* ≤ |arg(*ζ*)| ≤ *π* *and* |*ζ*| ≥ 0.

**Lemma 3** [12]. *Suppose that* *x*(*t*) = 0 *is an equilibrium point of the following system*

$$\begin{array}{c} D^{\alpha }x\left(t\right)=f\left(t,x\left(t\right)\right). \end{array}$$ (7)

*If there exist a Lyapunov function* *V*(*t*, *x*(*t*)) *and a class*-*K* *function* *g*_*i*_, *i* = 1, 2, 3 *such that*

$$\begin{array}{c} g_{1}\left(∥x\left(t\right)∥\right)\leq V\left(t,x\left(t\right)\right)\leq g_{2}\left(∥x\left(t\right)∥\right), \end{array}$$ (8)

$$\begin{array}{c} D^{\alpha }V\left(t,x\left(t\right)\right)\leq -g_{3}\left(∥x\left(t\right)∥\right), \end{array}$$ (9)

*then system* Eq (7) *is asymptotically stable*.

**Lemma 4** [13, 15]. *Let* $x\left(t\right)\in R^{n}$ *be a smooth function and* $G\in R^{n\times n}$ *be a positive definite matrix. Then it holds that*

$$\begin{array}{c} \frac{1}{2}D^{\alpha }x^{T}\left(t\right)Gx\left(t\right)\leq x^{T}\left(t\right)GD^{\alpha }x\left(t\right). \end{array}$$ (10)

### 2.2 Description of a fuzzy logic system

A fuzzy inference system contains four parts, i.e., the fuzzifier, the knowledge base, the fuzzy inference and the defuzzifier [17, 39–49]. The fuzzy rules are used by the fuzzy inference to construct a mapping from $x\left(t\right)={\left[x_{1}\left(t\right),x_{2}\left(t\right),\cdots ,x_{n}\left(t\right)\right]}^{T}\in R^{n}$ which is the input vector to an output $\hat{f}\left(t\right)\in R$. Suppose that there are N fuzzy rules are used. The *i*th rule can be expressed as

$$\begin{array}{c} \text{Rule}\;i:\text{if}\;x_{1}\left(t\right)\;\text{is}\;F_{1}^{i}\;\;\text{and}\cdots \;x_{n}\left(t\right)\;\text{is}\;F_{i}^{n}\;then\;\hat{f}\left(t\right)\;is\;g^{i}, \end{array}$$ (11)

where $F_{1}^{i},\cdots ,F_{n}^{i}$ represent fuzzy sets and *g*^*i*^ corresponds to the output of this fuzzy rule. The above fuzzy inference can be modeled by

$$\begin{array}{c} \hat{f}\left(x\left(t\right)\right)=\frac{\sum_{i=1}^{N}g^{i}\prod_{j=1}^{n}\mu_{F_{j}^{i}}\left(x_{j}\left(t\right)\right)}{\sum_{i=1}^{N}\prod_{j=1}^{n}\mu_{F_{j}^{i}}\left(x_{j}\left(t\right)\right)}, \end{array}$$ (12)

where $\mu_{F_{j}^{i}}\left(x_{j}\left(t\right)\right)$ is the value of membership of *x*_*j*_(*t*) to $F_{j}^{i}$. Denote $\vartheta^{T}\left(t\right)=\left[g^{1},g^{2},\cdots ,g^{N}\right]$ and *ϕ*(*x*(*t*)) = [*q*_1_(*x*(*t*)), *q*_2_(*x*(*t*)), ⋯, *q*_*N*_(*x*(*t*))]^*T*^, where *q*_*j*_ is defined by

$$\begin{array}{c} q_{j}=\frac{\prod_{j=1}^{n}\mu_{F_{j}^{i}}\left(x_{j}\left(t\right)\right)}{\sum_{i=1}^{N}\prod_{j=1}^{n}\mu_{F_{j}^{i}}\left(x_{j}\left(t\right)\right)}, \end{array}$$ (13)

then, we can rewrite Eq (12) as

$$\begin{array}{c} \hat{f}\left(x\left(t\right)\right)=\vartheta^{T}\left(t\right)\phi \left(x\left(t\right)\right). \end{array}$$ (14)

In fact, Eq (12) is the most frequently utilized in literatures. Based on the universal approximation theorem [39], we can use the fuzzy logic system Eq (12) to approximate any continuous function *f*(*t*) which is defined on some compact set *Ω* to an arbitrary degree of accuracy.

## 3 Main results

### 3.1 Description of fractional-order financial chaotic systems

The mathematical model of the fractional-order financial system to be considered in this paper is [9, 22, 23]

$$\begin{array}{c} \left\{ \begin{array}{c} D^{q}x_{1}\left(t\right)=x_{3}\left(t\right)+\left(x_{2}\left(t\right)-\alpha \right)x_{1}\left(t\right)\\ D^{q}x_{2}\left(t\right)=1-\beta x_{2}\left(t\right)-x_{1}^{2}\left(t\right)\\ D^{q}x_{3}\left(t\right)=-x_{1}\left(t\right)-\gamma x_{3}\left(t\right), \end{array}\right. \end{array}$$ (15)

where *α* corresponds to the saving amount, *β* represents the cost per investment, *γ* is the elasticity of demand of commercial market, and *q* ∈ (0, 1]. The first state variable *x*_1_(*t*), which represents the interest rate, can be effected by the surplus between investment and savings as well as structural adjustments of the prices. The second state variable *x*_2_(*t*) corresponds to the rate of investment, and inversely proportional to the cost of investment and the interest rate. The third state variable *x*_3_(*t*) depends on the difference between supply and demand in the market, and it can also be affected by the inflation rate. The equilibrium points of Eq (15) are:

$$\begin{array}{ccc} Q_{1} & = & (0,\frac{1}{\beta },0),\\ Q_{2} & = & (\sqrt{\frac{\gamma -\beta -\alpha \beta \gamma }{\gamma }},\frac{1+\alpha \gamma }{\gamma },-\frac{1}{\gamma }\sqrt{\frac{\gamma -\beta -\alpha \beta \gamma }{\gamma }}),\\ Q_{3} & = & (-\sqrt{\frac{\gamma -\beta -\alpha \beta \gamma }{\gamma }},\frac{1+\alpha \gamma }{\gamma },\frac{1}{\gamma }\sqrt{\frac{\gamma -\beta -\alpha \beta \gamma }{\gamma }}). \end{array}$$ (16)

With respect to the equilibrium point $Q^{*}={\left[x_{1}^{*},x_{2}^{*},x_{3}^{*}\right]}^{T}$, the Jacobian matrix is

$$\begin{array}{c} J_{Q}=[\begin{array}{ccc} -\alpha +x_{2}^{*} & x_{1}^{*} & 1\\ -2x_{1}^{*} & -\beta & 0\\ -1 & 0 & -\gamma \end{array}]. \end{array}$$ (17)

**Fig 1:**
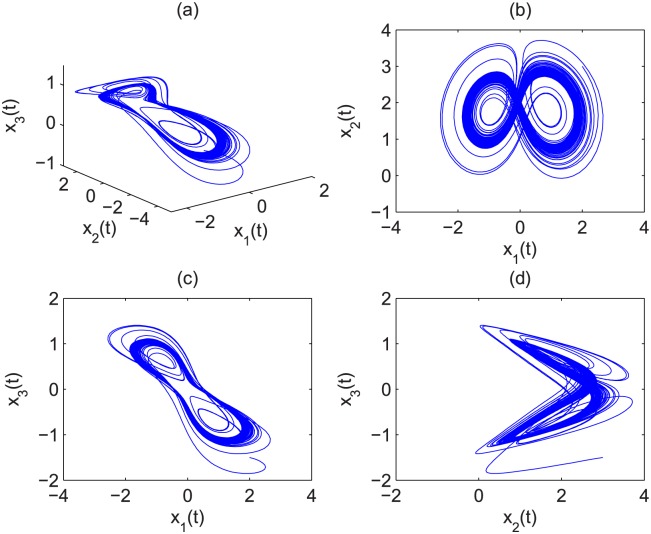
Chaotic behavior of fractional-order financial system Eq (15) with *q* = 0.86 in (a) *x*_1_(*t*) − *x*_2_(*t*) − *x*_3_(*t*), (b) *x*_1_(*t*) − *x*_2_(*t*), (c) *x*_1_(*t*) − *x*_3_(*t*) and (d) *x*_2_(*t*) − *x*_3_(*t*).

**Fig 2:**
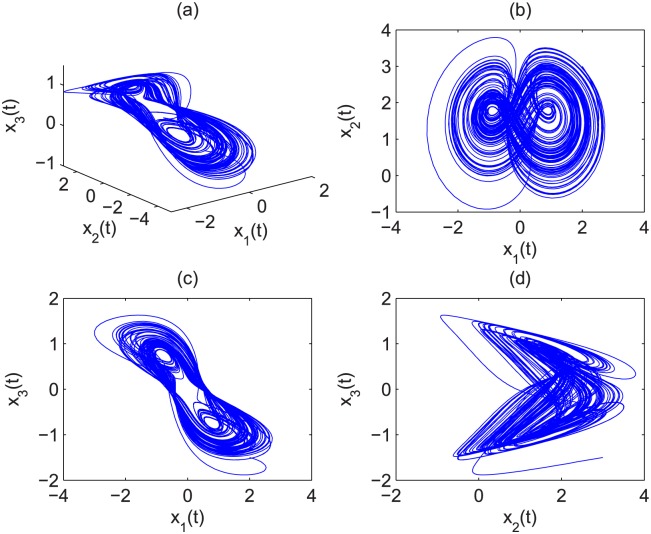
Chaotic behavior of fractional-order financial system Eq (15) with *q* = 0.97 in (a) *x*_1_(*t*) − *x*_2_(*t*) − *x*_3_(*t*), (b) *x*_1_(*t*) − *x*_2_(*t*), (c) *x*_1_(*t*) − *x*_3_(*t*) and (d) *x*_2_(*t*) − *x*_3_(*t*).

Suppose that *α* = 1, *β* = 0.1 and *γ* = 1, then the eigenvalues of the equilibrium *Q*_1_ = (0.000; 10.000; 0.000) are *ρ*_1_ = 8.8990, *ρ*_2_ = −0.8990, and *ρ*_3_ = −0.1000. As a result, it is a saddle point. For equilibrium points *Q*_2_ = (0.8944; 2.000; −0.8944) and *Q*_3_ = (−0.8944; 2.000; 0.8944), they are: *ρ*_1_ = −0.7609 and *ρ*_2,3_ = 0.3304 ± 1.4112*i*. Since they are unstable equilibriums, the condition for chaos is satisfied. It is easy to get the minimal commensurate order of the system is *q* > 0.8537. Let the initial conditions be *x*_1_(0) = 1, *x*_2_(0) = 2, *x*_3_(0) = −0.5. The chaotic attractor of the fractional-order financial system Eq (15) is shown in Figs 1 and 2 when *q* = 0.86 and *q* = 0.97, respectively.

### 3.2 Adaptive fuzzy controller design

Taking the system uncertainties and external disturbance into consideration, and let $A=[\begin{array}{ccc} -\alpha & 0 & 1\\ 0 & -\beta & 0\\ -1 & 0 & -\gamma \end{array}]$ and $f\left(x\left(t\right)\right)=[\begin{array}{c} -x_{1}\left(t\right)x_{2}\left(t\right)\\ 1-x_{1}^{2}\left(t\right)\\ 0 \end{array}]$, the controlled fractional-order financial chaotic system Eq (15) can be rewritten as

$$\begin{array}{c} D^{q}x\left(t\right)=Ax\left(t\right)+f\left(x\left(t\right)\right)+▵f\left(x\left(t\right)\right)+Gd\left(t\right)+GS\left(u\left(t\right)\right) \end{array}$$ (18)

where *x*(*t*) = [*x*_1_(*t*), *x*_2_(*t*), *x*_3_(*t*)]^*T*^ is the state vector, $G\in R^{3\times 3}$ is a constant control gain matrix, $u\left(t\right)={\left[u_{1}\left(t\right),u_{2}\left(t\right),u_{3}\left(t\right)\right]}^{T}\in R^{3}$ represents the control input to be designed, $S\left(u\left(t\right)\right)={\left[S\left(u_{1}\left(t\right)\right),S\left(u_{2}\left(t\right)\right),S\left(u_{3}\left(t\right)\right)\right]}^{T}\in R^{3}$ represents the input saturation, and △*f*(*x*(*t*)) and $d\left(t\right)\in R^{3}$ are the system uncertainty and external disturbance, respectively. In this paper, the input saturation is defined as

$$\begin{array}{c} S\left(u_{i}\left(t\right)\right)=\{\begin{array}{c} u_{i,max},\;u_{i}\left(t\right)\geq u_{i,max}\\ u_{i}\left(t\right),\;\;u_{i,min}\leq u_{i}\left(t\right)\leq u_{i,max}\\ u_{i,min},\;u_{i}\left(t\right)\leq u_{i,min} \end{array} \end{array}$$ (19)

where *u*_*i*,*max*_ > 0, *u*_*i*,*min*_ < 0, *i* = 1, 2, 3 are known constants. Define

$$\begin{array}{c} \omega_{i}\left(t\right)=\{\begin{array}{c} u_{i,max}-u_{i}\left(t\right),\;u_{i}\left(t\right)\geq u_{i,max}\\ 0,\;u_{i,min}\leq u_{i}\left(t\right)\leq u_{i,max}\\ u_{i}\left(t\right)-u_{i,min},\;u_{i}\left(t\right)\leq u_{i,min}. \end{array} \end{array}$$ (20)

Let *ω*(*t*) = [*ω*_1_(*t*), *ω*_2_(*t*), *ω*_3_(*t*)]^*T*^, then it follows from Eqs (19) and (20) that

$$\begin{array}{c} S(u(t))=u(t)+\omega (t). \end{array}$$ (21)

To proceed, the following Assumptions are needed.

**Assumption 1** *The control gain matrix G is an unknown positive definite matrix*.

**Assumption 2** *There exists an unknown positive constant* ${\bar{d}}_{i}$ *such that* $|d_{i}\left(t\right)|\leq {\bar{d}}_{i},i=1,2,3$.

Multiplying *G*^−1^ to both sides of Eq (18), denoting *P* = *G*^−1^ and using Eq (21), we have

$$\begin{array}{c} PD^{q}x\left(t\right)=\varpi \left(t\right)+d\left(t\right)+u\left(t\right) \end{array}$$ (22)

where ϖ(t)=PAx(t)+Pf(x(t))+P▵f(x(t))+ω(t) is an unknown nonlinear function. Denote $\varpi \left(t\right)={\left[\varpi_{1}\left(t\right),\varpi_{2}\left(t\right),\varpi_{2}\left(t\right)\right]}^{T}$.

Noting that the continuous nonlinear function $\varpi_{i}\left(t\right)$ is fully unknown, we can approximate it, by using the fuzzy logic system Eq (14), as

$$\begin{array}{c} {\hat{\varpi }}_{i}\left(t\right)=\vartheta_{i}^{T}\left(t\right)\phi_{i}\left(x\left(t\right)\right),i=1,2,3. \end{array}$$ (23)

The ideal parameter of *ϑ*_*i*_(*t*) can be defined as

$$\begin{array}{c} \vartheta_{i}^{*}=\text{arg}\min_{\vartheta_{i}\left(t\right)}[\underset{x(t)}{\sup}|\varpi_{i}\left(t\right)-{\hat{\varpi }}_{i}\left(t\right)|]. \end{array}$$ (24)

It is worth mentioning that the parameter $\vartheta_{i}^{*}$ is given only for theoretical analysis purpose. In fact, in the controller design procedure, we will not need its exact value. Let the parameter estimation error and the fuzzy system approximation error be

$$\begin{array}{c} {\tilde{\vartheta }}_{i}\left(t\right)=\vartheta_{i}\left(t\right)-\vartheta_{i}^{*}, \end{array}$$ (25)

and

$$\begin{array}{c} \varepsilon_{i}\left(t\right)=\varpi_{i}\left(t\right)-{\hat{\varpi }}_{i}\left(t\right), \end{array}$$ (26)

respectively. According to the results in [40, 42, 50], we can suppose that there exists an unknown positive constant ${\bar{\varepsilon }}_{i}$ such that

$$\begin{array}{c} |\varepsilon_{i}\left(t\right)|\leq {\bar{\varepsilon }}_{i}. \end{array}$$ (27)

Denote *ε*(*t*) = [*ε*_1_(*t*), *ε*_2_(*t*), *ε*_3_(*t*)]^*T*^, *ϑ*(*t*) = [*ϑ*_1_(*t*), *ϑ*_2_(*t*), *ϑ*_3_(*t*)], $\bar{\varepsilon }={\left[{\bar{\varepsilon }}_{1},{\bar{\varepsilon }}_{2},{\bar{\varepsilon }}_{3}\right]}^{T}$, $\tilde{\vartheta }\left(t\right)=\left[{\tilde{\vartheta }}_{1}\left(t\right),{\tilde{\vartheta }}_{2}\left(t\right),{\tilde{\vartheta }}_{3}\left(t\right)\right]$, $\vartheta^{*}={\left[\vartheta_{1}^{*},\vartheta_{2}^{*},\vartheta_{3}^{*}\right]}^{T}$, we have

$$\begin{array}{ccc} \hat{\varpi }\left(t\right)-\varpi \left(t\right) & = & \hat{\varpi }\left(t\right)-\hat{\varpi }\left(t,\vartheta^{*}\right)+\hat{\varpi }\left(t,\vartheta^{*}\right)-\varpi \left(t\right)\\ & = & \vartheta^{T}\left(t\right)\phi \left(x\left(t\right)\right)-\vartheta^{*T}\phi \left(x\left(t\right)\right)-\varepsilon \left(t\right)\\ & = & {\tilde{\vartheta }}^{T}\left(t\right)\phi \left(x\left(t\right)\right)-\varepsilon \left(t\right). \end{array}$$ (28)

To proceed, we will present the following two Lemmas.

**Lemma 5** *Let* z(t)∈R *be a smooth function. If* $D^{q}z\left(t\right)\leq 0$, *then the function* *z*(*t*) *is monotone decreasing*.

**Proof 1** *It is easy to get that*

$$\begin{array}{c} D^{q}z\left(t\right)+g\left(t\right)=0. \end{array}$$ (29)

*where* g(t)∈R *is a non-negative function. The Laplace transform of* Eq (29) *is*

$$\begin{array}{c} Z\left(s\right)=\frac{z(0)}{s}-\frac{G(s)}{s^{q}} \end{array}$$ (30)

*where* *Z*(*s*) *and* *G*(*s*) *represent the Laplace transforms of* *z*(*t*) *and* *g*(*t*), *respectively. The solution of* Eq (30) *can be given as*

$$\begin{array}{c} z\left(t\right)=z\left(0\right)-D^{-q}g\left(t\right). \end{array}$$ (31)

*Noting that* *g*(*t*) ≥ 0 *for all* *t* > 0, *according to* Eq (1) *we have* $D^{-q}g\left(t\right)\geq 0$. *Thus, it follows* Eq (31) *that* *z*(*t*) ≤ *z*(0), *and the function* *z*(*t*) *is monotone decreasing*.

**Lemma 6** *Let* $V_{1}\left(t\right)=\frac{1}{2}z_{1}^{2}\left(t\right)+\frac{1}{2}z_{2}^{2}\left(t\right)$, *where* $z_{1}\left(t\right)\in R$ *and* $z_{2}\left(t\right)\in R$ *are smooth functions. Suppose that*

$$\begin{array}{c} D^{q}V_{1}\left(t\right)\leq -\kappa z_{1}^{2}\left(t\right) \end{array}$$ (32)

*where* *κ* > 0. *Thus, it holds that*

$$\begin{array}{c} z_{1}^{2}\left(t\right)\leq 2V_{1}\left(0\right)E_{q}\left(-2\kappa t^{q}\right). \end{array}$$ (33)

**Proof.** Taking the *q*-th fractional integral Eq (32) gives

$$\begin{array}{c} V_{1}\left(t\right)-V_{1}\left(0\right)\leq -\kappa D^{-q}z_{1}^{2}\left(t\right). \end{array}$$ (34)

Then Eq (34) implies

$$\begin{array}{c} z_{1}^{2}\left(t\right)\leq 2V_{1}\left(0\right)-2\kappa D^{-q}x^{2}\left(t\right). \end{array}$$ (35)

Thus we know that we can find a function *h*(*t*) ≥ 0 such that

$$\begin{array}{c} z_{1}^{2}\left(t\right)+h\left(t\right)=2V_{1}\left(0\right)-2\kappa D^{-q}z_{1}^{2}\left(t\right). \end{array}$$ (36)

Then the Laplace transform (L{·}) of Eq (36) is

$$\begin{array}{c} Z\left(s\right)=2V_{1}\left(0\right)\frac{s^{q-1}}{s^{q}+2\kappa }-\frac{s^{q}}{s^{q}+2\kappa }H\left(s\right). \end{array}$$ (37)

Based on Eq (4), we can solve Eq (37) as

$$\begin{array}{c} z_{1}^{2}\left(t\right)=2V_{1}\left(0\right)E_{q}\left(-2\kappa t^{q}\right)-2h\left(t\right)*\left[t^{-1}E_{q,0}\left(-2\kappa t^{q}\right)\right] \end{array}$$ (38)

where * represents the convolution operator. It is easy to know that both *E*_*q*, 0_(−2*kt*^*q*^) and *t*^−1^ are nonnegative functions, thus we have that Eq (33) holds. This ends the proof of Lemma 6.

According to Lemma 6, we can obtain the following results.

**Lemma 7** *Suppose that* $V_{2}\left(t\right)=\frac{1}{2}z^{T}\left(t\right)G_{1}z\left(t\right)+\frac{1}{2}r^{T}\left(t\right)G_{2}r\left(t\right)$, *where* $z\left(t\right)\in R^{n}$ *and* $r\left(t\right)\in R^{n}$ *are smooth functions, and* *G*_1_ *and* $G_{2}\in R^{n\times n}$ *are two positive definite matrices. Then, if there exists a positive definite matrix* *G*_3_ *and such that*

$$\begin{array}{c} D^{q}V_{2}\left(t\right)\leq -x^{T}\left(t\right)G_{3}x\left(t\right), \end{array}$$ (39)

*then we have* ‖*x*(*t*)‖ *and* ‖*y*(*t*)‖ *will converge to the origin asymptotically (i.e.* $\lim_{t\to \infty }∥x\left(t\right)∥=0$).

The main results can be concluded as the following Theorem.

**Theorem 1** *Consider the controlled fractional-order financial chaotic system* Eq (18) *under the Assumptions 1 and 2. Suppose that the controller is designed as*

$$\begin{array}{c} u\left(t\right)=-\vartheta^{T}\left(t\right)\phi \left(x\left(t\right)\right)-K_{1}x\left(t\right)-{\hat{K}}_{2}\left(t\right)\text{sgn}\left(x\left(t\right)\right), \end{array}$$ (40)

*where* *K*_1_ = diag(*k*_1*i*_, *k*_12_, *k*_13_) *and* ${\hat{K}}_{2}\left(t\right)=\text{diag}\left({\hat{k}}_{21}\left(t\right),{\hat{k}}_{22}\left(t\right),{\hat{k}}_{23}\left(t\right)\right)$ (*k*_1*i*_ > 0 *is positive design parameter and* ${\hat{k}}_{2i}\left(t\right)$ *is adjustable parameter which is the estimation of the unknown constant* $k_{2i}={\bar{d}}_{i}+{\bar{\varepsilon }}_{i}$) *and the fractional adaptation laws are given as*

$$\begin{array}{c} D^{\alpha }\vartheta_{i}^{T}\left(t\right)=\delta_{1i}x_{i}\left(t\right)\phi \left(t\right) \end{array}$$ (41)

*and*

$$\begin{array}{c} D^{\alpha }k_{2i}\left(t\right)=\delta_{2i}\left|x_{i}\left(t\right)\right| \end{array}$$ (42)

*where* *δ*_1*i*_ *and* *δ*_2*i*_ *are positive design parameters, then we have that the state variables will converge to the origin asymptotically and all signals involved will keep bounded*.

**Proof 2** *From* Eqs (22), (28) *and* (40) *we have*

$$\begin{array}{ccc} PD^{q}x\left(t\right) & = & \varpi \left(t\right)+d\left(t\right)-\vartheta^{T}\left(t\right)\phi \left(x\left(t\right)\right)-K_{1}x\left(t\right)-K_{2}\text{sgn}\left(x\left(t\right)\right)\\ & = & d\left(t\right)-K_{1}x\left(t\right)-K_{2}\text{sgn}\left(x\left(t\right)\right)-{\tilde{\vartheta }}^{T}\left(t\right)\phi \left(t\right)+\varepsilon \left(t\right). \end{array}$$ (43)

*Multiplying* *x*^*T*^(*t*) *to both sides of* Eq (43), *and using Assumptions* 1 *and* 2, *we can obtain*

$$\begin{array}{ccc} x^{T}\left(t\right)PD^{q}x\left(t\right) & = & \sum_{i=1}^{3}d_{i}\left(t\right)x_{i}\left(t\right)-x^{T}\left(t\right)K_{1}x\left(t\right)-\sum_{i=1}^{3}{\hat{k}}_{2i}\left(t\right)\left|x_{i}\left(t\right)\right|\\ & & -\sum_{i=1}^{3}x_{i}\left(t\right){\tilde{\vartheta }}_{i}^{T}\left(t\right)\phi_{i}\left(t\right)+\sum_{i=1}^{3}\varepsilon_{i}\left(t\right)x_{i}\left(t\right)\\ & \leq & \sum_{i=1}^{3}{\bar{d}}_{i}|x_{i}\left(t\right)|-x^{T}\left(t\right)K_{1}x\left(t\right)-\sum_{i=1}^{3}{\hat{k}}_{2i}\left(t\right)\left|x_{i}\left(t\right)\right|\\ & & -\sum_{i=1}^{3}x_{i}\left(t\right){\tilde{\vartheta }}_{i}^{T}\left(t\right)\phi_{i}\left(t\right)+\sum_{i=1}^{3}{\bar{\varepsilon }}_{i}\left|x_{i}\left(t\right)\right|\\ & = & \sum_{i=1}^{3}k_{2i}|x_{i}\left(t\right)|-x^{T}\left(t\right)K_{1}x\left(t\right)-\sum_{i=1}^{3}{\hat{k}}_{2i}\left(t\right)\left|x_{i}\left(t\right)\right|\\ & & -\sum_{i=1}^{3}x_{i}\left(t\right){\tilde{\vartheta }}_{i}^{T}\left(t\right)\phi_{i}\left(t\right)\\ & = & -x^{T}\left(t\right)K_{1}x\left(t\right)-\sum_{i=1}^{3}x_{i}\left(t\right){\tilde{\vartheta }}_{i}^{T}\left(t\right)\phi_{i}\left(t\right)-\sum_{i=1}^{3}{\tilde{k}}_{2i}\left(t\right)\left|x_{i}\left(t\right)\right| \end{array}$$ (44)

*where*

$$\begin{array}{c} {\tilde{k}}_{2i}\left(t\right)={\hat{k}}_{2i}\left(t\right)-k_{2i} \end{array}$$ (45)

*is the estimation error*.

*Let*

$$\begin{array}{c} V\left(t\right)=\frac{1}{2}x^{T}\left(t\right)Px\left(t\right)+\sum_{i=1}^{3}\frac{1}{2\delta_{1i}}{\tilde{\vartheta }}_{i}^{T}\left(t\right){\tilde{\vartheta }}_{i}\left(t\right)+\sum_{i=1}^{3}\frac{1}{2\delta_{2i}}{\tilde{k}}_{2i}^{2}\left(t\right). \end{array}$$ (46)

*Noting that* $D^{q}{\tilde{k}}_{2i}\left(t\right)=D^{q}{\hat{k}}_{2i}\left(t\right)$ *and* $D^{q}\tilde{\vartheta }\left(t\right)=D^{q}\hat{\vartheta }\left(t\right)$. *Thus, Lemma* 4, Eqs (41), (42) *and* (44) *imply that*

$$\begin{array}{ccc} D^{q}V\left(t\right) & \leq & x^{T}\left(t\right)PD^{q}x\left(t\right)+\sum_{i=1}^{3}\frac{1}{\delta_{1i}}{\tilde{\vartheta }}_{i}^{T}\left(t\right)D^{q}{\tilde{\vartheta }}_{i}\left(t\right)+\sum_{i=1}^{3}\frac{1}{\delta_{2i}}{\tilde{k}}_{2i}\left(t\right)D^{q}{\tilde{k}}_{2i}\left(t\right)\\ & = & -x^{T}\left(t\right)K_{1}x\left(t\right)-\sum_{i=1}^{3}x_{i}\left(t\right){\tilde{\vartheta }}_{i}^{T}\left(t\right)\phi_{i}\left(t\right)-\sum_{i=1}^{3}{\tilde{k}}_{2i}\left(t\right)\left|x_{i}\left(t\right)\right|\\ & & +\sum_{i=1}^{3}\frac{1}{\delta_{1i}}{\tilde{\vartheta }}_{i}^{T}\left(t\right)D^{q}{\tilde{\vartheta }}_{i}\left(t\right)+\sum_{i=1}^{3}\frac{1}{\delta_{2i}}{\tilde{k}}_{2i}\left(t\right)D^{q}{\tilde{k}}_{2i}\left(t\right)\\ & = & -x^{T}\left(t\right)K_{1}x\left(t\right)-\sum_{i=1}^{3}x_{i}\left(t\right){\tilde{\vartheta }}_{i}^{T}\left(t\right)\phi_{i}\left(t\right)-\sum_{i=1}^{3}{\tilde{k}}_{2i}\left(t\right)\left|x_{i}\left(t\right)\right|\\ & & +\sum_{i=1}^{3}\frac{1}{\delta_{1i}}{\tilde{\vartheta }}_{i}^{T}\left(t\right)D^{q}{\hat{\vartheta }}_{i}\left(t\right)+\sum_{i=1}^{3}\frac{1}{\delta_{2i}}{\tilde{k}}_{2i}\left(t\right)D^{q}{\hat{k}}_{2i}\left(t\right)\\ & = & -x^{T}\left(t\right)K_{1}x\left(t\right). \end{array}$$ (47)

*Noting that* $D^{q}V\left(t\right)\leq 0$, *according to Lemma* 5 *we know that* *x*(*t*), ${\tilde{\vartheta }}_{i}\left(t\right)$ *and* ${\tilde{k}}_{2i}\left(t\right)$ *will keep bounded, and as a result*, *ϑ*_*i*_(*t*) *and* ${\hat{k}}_{2i}\left(t\right)$ *are all bounded for all* *t* ≥ 0. *Thus, we know that all signals involved will remain bounded. Besides, it follows from Lemma* 7 *and* Eq (47) *that* *x*(*t*) *will converge to zero asymptotically. And this completes the proof of Theorem* 1.

**Remark 1** *It is worth mentioning that in the controller design, the model of the fractional-order financial chaotic systems is not needed (see,* Eq (40)). *Besides, the proposed method can be generalized to control many other uncertain fractional-order nonlinear systems*.

**Remark 2** *In* Eq (40), *the sign function, which may result in the chattering phenomenon, is used. To solve this problem, we can use some continuous function to replace it*.

**Remark 3** *It should be noted that the input saturation in fractional-order systems is also considered in* [37] *and* [38]. *In* [37], *stability and stabilization for a class of fractional-order linear system is discussed. To handle the effect of the input saturation, a memoryless nonlinearity is used, thus the input saturation can be written as a linear matrix inequality. This method is very interesting and easy to be realized. However, the results of this work can be guaranteed only is a small region of the initial condition. In* [38], *a fractional-order nonlinear model is considered. Just like the work of* [37], *a memoryless nonlinearity is also used. Besides, to discuss the stability of the closed-loop system, a assumption*, $\lim_{x\to 0}\frac{f(x(t))}{x(t)}=0$ *is used to impose restrictions on the system nonlinear function. However, this is a very restrictive condition, which is very hard to be satisfied in real physical systems. As a comparison, the above mentioned problems will not occur in this paper*.

## 4 Simulation studies

In the simulation, the system parameters are chosen as that in Section 3, i.e., *α* = 1, *β* = 0.1 and *γ* = 1. The fractional-order *q* is selected as *q* = 0.91, and the initial condition is chosen as *x*(0) = [2, −3, 3]^*T*^. The design parameters are *k*_1*i*_ = 0.8, *δ*_1*i*_ = *δ*_2*i*_ = 1, *i* = 1, 2, 3, and the initial condition for ${\hat{k}}_{1i}\left(t\right)$ is ${\hat{k}}_{1i}\left(0\right)=1$. The control gain matrix is *G* = diag(0.7, 1.1, 1), which is positive definite. The system uncertainty is $▵f\left(x\left(t\right)\right)={\left[0.1x_{1}\left(t\right)x_{2}\left(t\right),-1-0.2x_{3}^{2}\left(t\right),\sin\left(x_{1}\left(t\right)\right)\right]}^{T}$, and the external disturbance is *d*(*t*) = [0.1sin(*t*), 0.1cos(*t*), 0.15sin(3*t*)].

For the fuzzy logic systems, we choose four Gaussian membership functions distributed on [−3, 3]. As a results, there are 4 × 4 × 4 = 64 fuzzy rules are involved in the controller design. The initial condition is $\theta_{i}\left(0\right)=0\in R^{64},i=1,2,3$.

**Fig 3:**
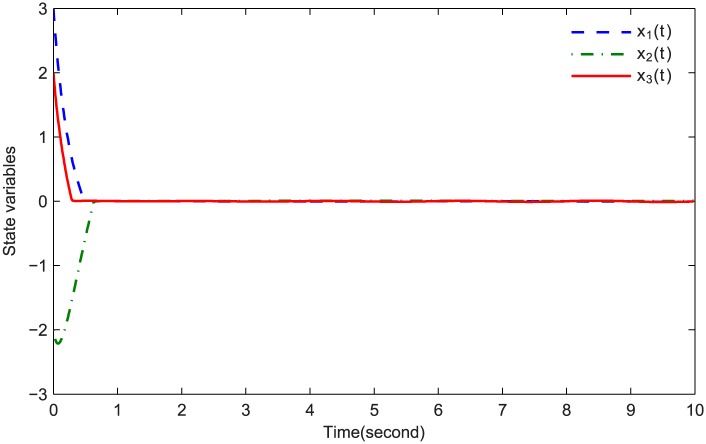
Time responses of the states variables.

**Fig 4:**
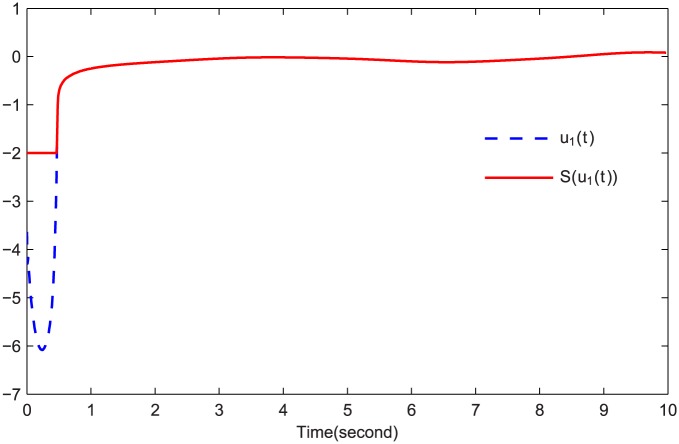
*u*_1_(*t*) and $S(u_{1}\left(t\right))$.

**Fig 5:**
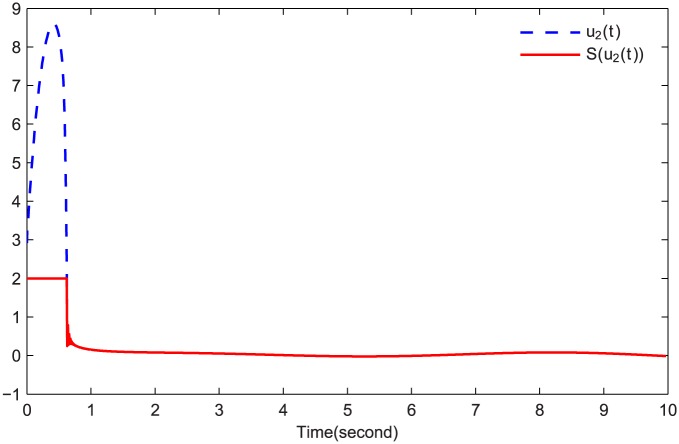
*u*_2_(*t*) and $S(u_{2}\left(t\right))$.

**Fig 6:**
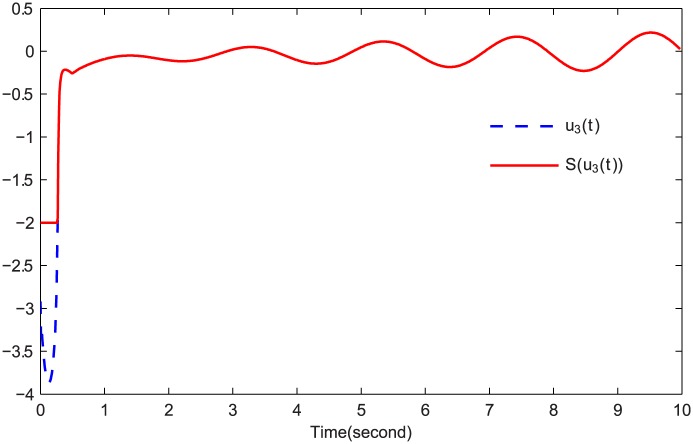
*u*_3_(*t*) and $S(u_{3}\left(t\right))$.

**Fig 7:**
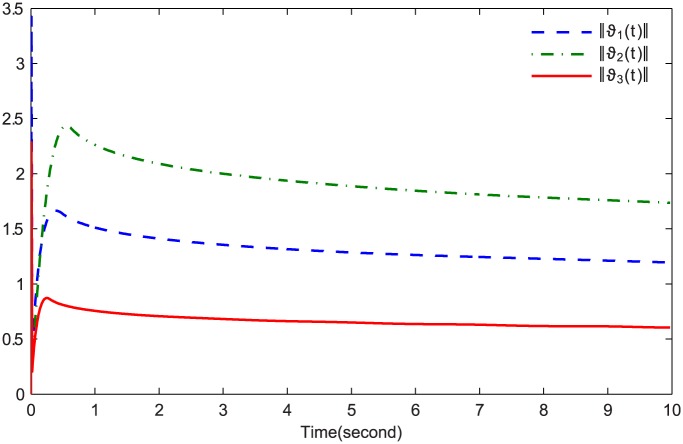
‖*ϑ*_1_(*t*)‖, ‖*ϑ*_2_(*t*)‖ and ‖*ϑ*_3_(*t*)‖.

**Fig 8:**
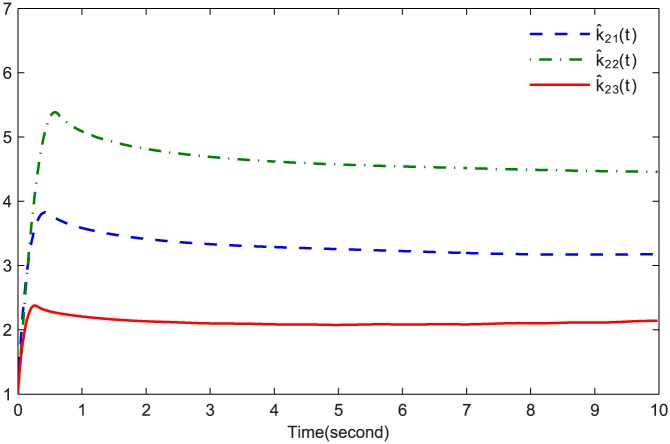
${\hat{k}}_{21}\left(t\right)$, ${\hat{k}}_{22}\left(t\right)$ and ${\hat{k}}_{23}\left(t\right)$.

The simulations are presented in Figs 3–8. The state variables, which is indicated in Fig 3, tend to the origin rapidly. To eliminate the chattering phenomenon which is produced by the sign(⋅) function in Eq (40), we use the continuous function arctan(10) to replace it. The control inputs and their input saturations are shown in Figs 4–6. The norm of the fuzzy parameters, and the estimations of the unknown constants, are given in Figs 7 and 8, respectively. It can be seen that the state variables have a rapid convergence, and the adaptive fuzzy controller works well even in a noisy environment with input saturation as well as a fully unknown system model. Yet, the state variables cannot stop at the origin but have some tiny fluctuations near the origin. The reasons for this phenomenon are: (1) with respect to a fractional-order nonlinear system, whenever the system trajectories reach the equilibrium point, they can not stay there thereafter because there are no finite-time stable equilibria in fractional-order systems [51]; (2) sign(⋅) is replaced by arctan(10) in this paper so that asymptotical convergence of the tracking error cannot be guaranteed.

**Fig 9:**
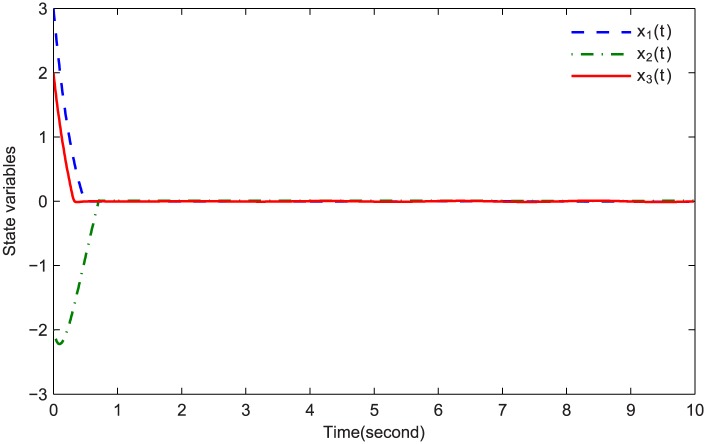
Simulation results for *q* = 0.98: State variables.

**Fig 10:**
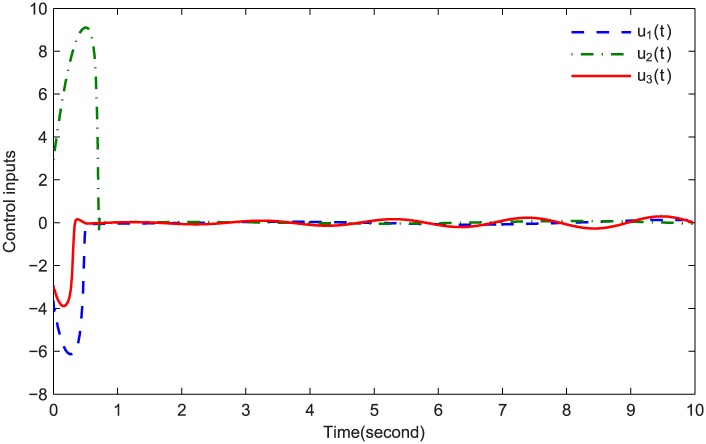
Simulation results for *q* = 0.98: Control inputs.

It should be pointed out that the proposed methods is valid for all *q* ∈ (0, 1]. The simulation results for *q* = 0.98 are given in Figs 9 and 10, from which we can see that the results are the same as that of the case *q* = 0.91.

## 5 Conclusion

Most of real world systems are inflicted by input constraints, especially in financial systems. This paper provides an adaptive fuzzy controller for uncertain fractional-order financial chaotic systems subject to input saturation. The saturation is divided into two parts, i.e., an unknown nonlinear function and the control input to be determined. The unknown part together with system uncertainty is approximated by a fuzzy logic system. It is showed that fractional-order adaptation laws can be given to eliminate the fuzzy approximation errors as well as estimation errors. How to design adaptive fuzzy control for fractional-order financial chaotic system (commensurate or incommensurate) with other kinds of input nonlinearities, such as backlash-like hysteresis and dead-zones, is one of our future research directions.
